# Effect of Graphite Filler Type on the Thermal Conductivity and Mechanical Behavior of Polysulfone-Based Composites

**DOI:** 10.3390/polym14030399

**Published:** 2022-01-20

**Authors:** Hussam Mohammad, Andrey A. Stepashkin, Victor V. Tcherdyntsev

**Affiliations:** Laboratory of Functional Polymer Materials, National University of Science and Technology “MISIS”, Leninskii Prosp. 4, 119049 Moscow, Russia; hussam.mhd22@gmail.com (H.M.); a.stepashkin@misis.ru (A.A.S.)

**Keywords:** polysulfone, composites, natural graphite, artificial graphite, expanded graphite, flexural strength, thermal conductivity

## Abstract

The goal of this study was to create a high-filled composite material based on polysulfone using various graphite materials. Composite material based on graphite-filled polysulfone was prepared using a solution method which allows the achievement of a high content of fillers up to 70 wt.%. Alongside the analysis of the morphology and structure, the thermal conductivity and mechanical properties of the composites obtained were studied. Structural analysis shows how the type of filler affects the structure of the composites with the appearance of pores in all samples which also has a noticeable effect on composites’ properties. In terms of thermal conductivity, the results show that using natural graphite as a filler gives the best results in thermal conductivity compared to artificial and expanded graphite, with the reduction of thermal conductivity while increasing temperature. Flexural tests show that using artificial graphite as a filler gives the composite material the best mechanical load transfer compared to natural or expanded graphite.

## 1. Introduction

Over millennia, man has interacted with a range of materials, from the stone age to the iron age, and now to the polymer age which is considered the most significant technological achievement of the millennium. In these days polymers and other materials like metals and ceramics are used in a variety of home and industrial applications in medical, aerospace and aviation sectors due to their light weight, simplicity of manufacture, flexibility, and low manufacturing costs [1,2]. However, these materials’ poor electrical and thermal conductivity has limited their use. When compared to more traditional materials, new polymer-based materials known as composite materials have gained popularity in high-performance applications. Fillers like carbon and metal powders have been widely employed to improve the electrical and thermal conductivity of insulating polymers, resulting in their wide use in industries including energy, electronics, airplanes, and vehicles. In addition to the superior electrical and mechanical capabilities of these materials, they are used in devices such as touch screen displays, health monitoring sensors, functional clothing, and other smart textile items [3,4,5,6,7]. Also, conductive composite materials these days have been developed to overcome the problems of lightning strikes and ice accumulation, rather than employing metals, which have various drawbacks such as heaviness and corrosion of the composite material used in airplane construction [8,9].

Carbon fillers in polymer composites have attracted scientific and industrial attention in the recent decade, thanks to their increasing technological potential in a variety of applications. The well-known reinforcement effect of carbon fillers in polymer composites is linked to improved electrical and thermal conductivity as well as mechanical properties of matrix polymers. Many studies have been published on the structural and physical properties of polymer composites containing various types of carbon that range significantly in size, geometry, and physical properties, such as carbon nanotubes, graphene, carbon black, natural and artificial graphite, and so on [10,11,12]. Extensive research has been conducted to study the feasibility of using polymeric compounds modified with micrometric materials for application in various fields of industry due to the increasing need for lightweight products with superior thermal and mechanical capabilities [11,12,13,14]. 

Many types of filler were recently reported to be integrated into a polymer matrix to synthesize thermal interface materials. It was noticed that the thermal conductivity of composites grew slowly as filler content increased until the filler percentage hit a percolation threshold, at which point a fast increase in conductivity began. Unfortunately, substantial micro-filler loadings (>50 wt.%) are commonly required to endow composites with exceptional thermal conductivity exceeding 5 W/m·K and, as a result, the mechanical and processing qualities will encounter some obstacles. Although nanofillers outperform conventional fillers in terms of increasing heat conductivity at low filler concentrations (less than 10 wt.%), their agglomeration tendency at higher filler concentrations due to robust self-interaction limits their application [15,16,17].

Carbon nanoparticles have recently been actively used as fillers for thermoplastic matrices, including polymethyl methacrylate [18], polypropylene [19,20,21], ethylene vinyl acetate [22], polyamide [23,24], polyurethane [25], polysulfone [26,27], polyethersulfone [28] and other thermoplastics. The types and structures of carbon nanofillers are demonstrated to have a considerable impact on the characteristics of the resulting composite materials; however, evidence on the nature of this effect is conflicting. For example, a study comparing the efficiency of carbon nanofibers, carbon black, graphite, and carbon nanotubes in the formation of electrically conductive networks in a polymer matrix found that nanofibers had the highest efficiency [22], while another study comparing carbon nanofibers, carbon black, and graphite found that carbon black had an advantage [22]. The effect of various shapes of carbon black nanoparticles was studied in [21], and it was discovered that particles with a branching shape build an electrically conductive framework more efficiently than particles with a spherical shape. The most effective construction of a conducting frame occurs as a synergistic effect of the interaction of several types of carbon filler, according to studies [17,23,24,28]. Carbon fillers have a good effect on the rheological features [23], mechanical properties [16,19,26], and fire resistance [25] of polymer composites, in addition to their electrically conductive capabilities.

Thermal conductivity via phonons in nano carbons, such as graphene, graphite, carbon fiber, and carbon nanotubes, is known to be theoretically limited at room temperature. When measured at room temperature, for example, the thermal conductivity of single-layer graphene can be as high as 4000–7000 W/m·K [29,30,31].

Graphite is a highly conductive crystalline form of carbon that occurs naturally or is synthesized. Graphite is heated when an electric current is passed through it. However, because of their high aspect ratio and theoretical thermal conductivity (129 W/m·K), natural graphite flakes have attracted a lot of attention as platelet-like thermally conductive fillers [31]. Another form of graphite is expanded graphite, commonly known as “exfoliated” graphite, which could be a useful filler in the production of conductive polymers. Exfoliated graphite is a new conducting filler with a layered structure that has unique electrical and structural properties. It is used in aerospace and automotive applications. When expanded graphite is mixed into a polymer matrix, the conducting composite materials acquire outstanding thermal and electrical conductivity as well as good mechanical properties, owing to its wide surface area and high aspect ratio. In comparison to other carbon-based fillers such as carbon nanotubes and nanofibers, one of the most attractive aspects of EG is its low cost and processability, which makes it ideal for the development of composites reinforced with it [32,33].

Nowadays, using such polymers as poly ether ether kethone, polysulfone and polyphenylene sulfone to create composite materials, which have high heat resistance, high operating temperatures, and good strength characteristics has attracted many researchers. An increase in their thermal conductivity makes it possible to significantly expand the area of their application. Several thermoplastic-based carbon-reinforced composites have been developed and studied in recent years. Due to their heat stability and good mechanical qualities, high-performance polymers are of special importance among thermoplastics such as polysulfone (PSU). PSU is widely used nowadays because of its remarkable properties: thermal properties, mechanical properties, chemical stability, great strength and toughness, and the ability to obtain polymeric membranes with various applications. PSU’s high-temperature capability allows it to be used in applications where other polymeric materials fail [34,35,36].

The use of traditional methods for obtaining filled polymer materials, such as extrusion mixing, does not allow highly filled compositions to be obtained due to their high viscosity, and the filling limits in this case are values of the order of 20–30 wt.%. The method we propose for obtaining materials using mortar technology can significantly increase degrees of filling up to 70 wt.%. The aim of the present paper was to study the effect of graphite fillers on the thermal and mechanical properties of the polysulfone-based composites using a high content of fillers up to 70 wt.%.

## 2. Materials and Methods

### 2.1. Materials

In the present study, the matrix material used was polysulfone (PSU) Ultrason S 2010 (BASF, Ludwigshafen, Germany), which is used to manufacture various parts of the power pack in modern civil aircraft. To prepare thermally and electrically conductive composite, different types of graphite were used as fillers: artificial graphite AG (grand GMZ, Moscow Electrode Plant, Moscow, Russia), natural graphite NG (GL-1 GOST 5279–74—Taiginsky GOK, Kyshtym, Russia) and thermoexpanded graphite, EG. EG was obtained by the authors from NG by a chemical method with subsequent heat treatment at a high temperature as described in Section 2.2.

### 2.2. Synthesize and Composite Preparation

Intercalation was performed using standard technology to produce thermally expanded graphite as follows. After filling a glass beaker with 100 mL of sulfuric acid and under continuous stirring, samples of natural graphite (3 g) and (5 g) of potassium dichromate (K_2_Cr_2_O_7_) were added to the glass beaker at room temperature for 3 h. While the stirring process continued, 100 mL of hydrogen peroxide was added to the solution to obtain an efficient exfoliation of graphite and the oxidation process continued for about 20 min. The solution was filtered after and washed by several liters of distilled water until the pH value of the compound was natural. Graphite flake’s crystalline structure is made up of stacked planes of carbon atoms organized in a hexagonal pattern. Within the basal planes, there is strong covalent bonding and weak van der Waals bonding between the layers. Graphite intercalation compounds (GIC) can be made with a variety of molecules, atoms, and ions placed between the carbon planes in the graphite crystal structure. Therefore, the GIC was prepared after adding the distilled water. The GIC was dried in the dryer for 5 h at 160 °C. At the final stage, the GIC was exposed to high-temperature thermal shock at about 800 °C which caused the vaporization of the GIC, and this caused an expansion in volume with the c-direction compared to natural graphite flakes. At the end of thermal treatment, the EG was obtained.

The composite material we prepared using the solution casting method, which allowed us to use high concentrations of fillers. The polymeric solution was prepared from polysulfone in N-methylpyrrolidone (CAS: 872-50-4, Molar mass: 99.13 g/mol, Empirical Formula C_5_H_9_NO) is obtained with a polymer concentration of 20–40 wt.%, and mechanically stirred for 24 h using a magnetic stirrer. After that, a filler is introduced into the resulting solution and using an overhead stirrer, homogeneous mixtures of polymer with fillers with concentrations from 30 to 70 wt.% with a step of 10 wt.% were obtained. The resultant material was utilized to prepare the samples by hot pressing and forming them into disks (diameter 12.7 mm, thickness 2 mm) for thermal analysis and into cuboid shape (40 × 10 × 2) for mechanical analysis once the solvent had evaporated.

### 2.3. Characterization

The size and morphology of the particles have a significant effect on dispersion efficiency and stability, so the fillers were investigated by scanning electron microscopy (SEM) (Hitachi TM-1000 Hitachi Ltd., Tokyo, Japan). The particle size measurements of the fillers were carried out by two methods using a laser particle size analyzer, and according to the data of scanning electron microscopy using a Hitachi TM-1000 microscope (Hitachi Ltd., Tokyo, Japan) using Origin lab program (Northampton, MA, USA). X-ray diffraction (XRD) (Rigaku, Japan) maximum power of 12 kW, voltage of 20–60 kV, current of 10–200 mA. Raman spectroscopy Thermo DXR (USA) was conducted with wavelengths of 532 and 780 nm and maximum power of 10 mW and 24 mW, respectively.

The LFA 447 Nanoflash (Netzsch-Gerätebau GmbH, Selb, Germany) diffusivity device was used to measure the thermal conductivity of the additive-filled polymers, which is based on a xenon flash lamp and conforms to ASTM E1461. A single xenon flash tube pulse is used to irradiate a test sample uniformly on one side. Using an infrared detector, the temperature rise on the other side is monitored as a function of time. The thermal diffusivity can be computed immediately based on the geometry of the temperature rise curve.

To study the mechanical properties of preparing composite material, samples were investigated using ZWICK/ROELL Z020 (Group, Ulm, Germany), with working space (W × H) 440 × 1795 mm and traverse speed 10 mm/min in the entire load range.

## 3. Results and Discussion

### 3.1. Structure of Graphite Powders

#### 3.1.1. Morphology and Microstructure

**Figure 1:**
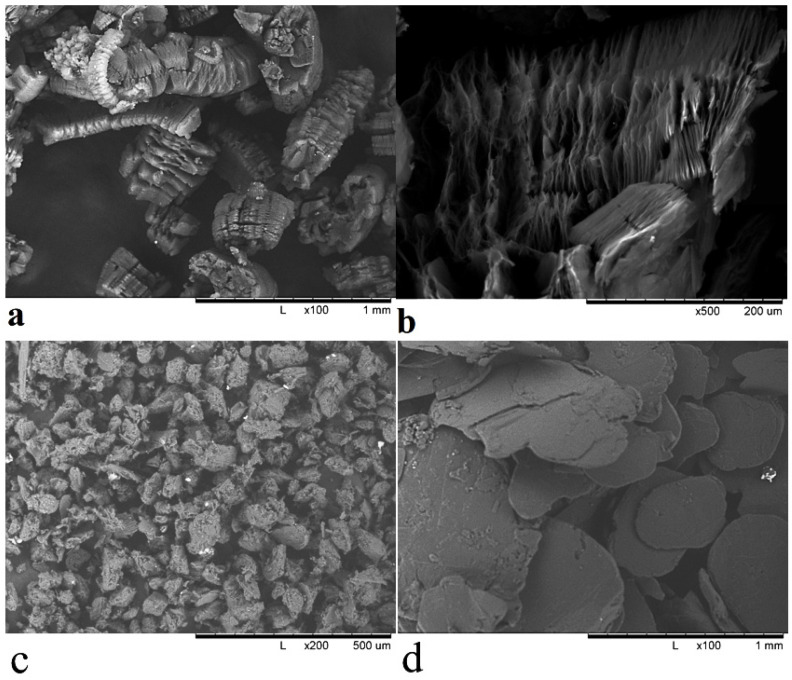
Scanning electron microscopy (SEM) images of: thermoexpanded graphite (EG) (**a**,**b**); artificial graphite (AG) (**c**); natural graphite (NG) (**d**).

The morphology and microstructure of expanded graphite are shown in Figure 1. Expanded graphite structure is like all-natural exfoliated graphene sheets as shown in Figure 1a with particle size about 120 μm. Graphite nanosheets are thin graphite flake layers with smooth and consistent surfaces. After chemical treatment, the chips are created in layers. Random deformation of parallel sheets of sectioned graphite produces EG. In our research, it shows rather large stacks of densely compacted EG particles with a worm-shape structure and a significant increase in interlayer distance when compared to natural graphite interlayer spacing. As the intercalating agent decomposes, therefore, instantaneous thrust acting in the axial direction (normal direction) of the graphite sheets is initiated, and the graphite layers are pushed open sufficiently to form expanded graphite particles. However, the gas generated by the decomposition of the interlayer intercalating agents could not completely open the graphite layers and that is why the edges of the layers are almost closed, as shown in Figure 1b,c shows the artificial graphite under microscope which appears like a small particle with a diameter of about 50 μm in addition to having some pores in the particles. Natural graphite is shown in Figure 1d, and looks like it is made up of large sheets with smooth and durable surfaces with a particle size of about 200 to 500 μm.

#### 3.1.2. Particle Size Analysis

**Figure 2:**
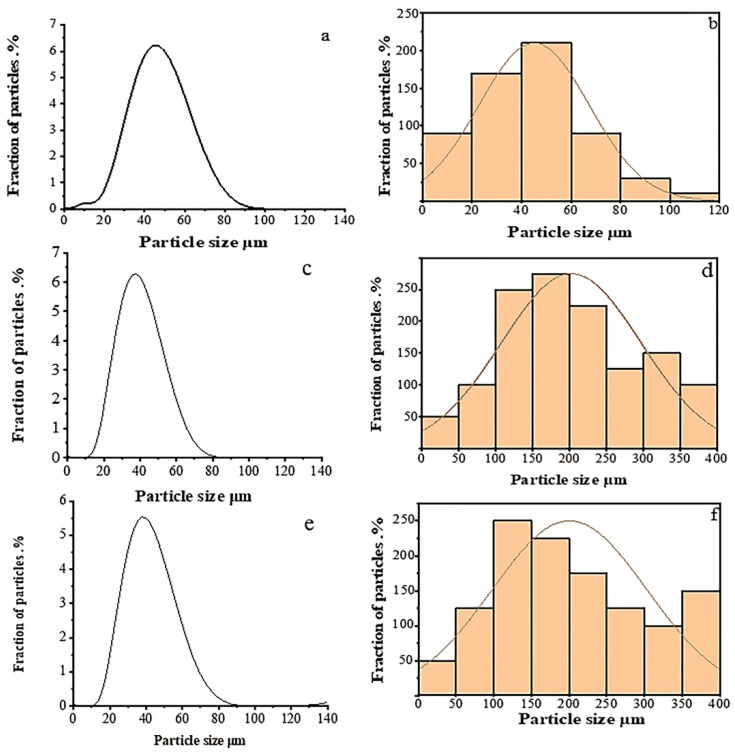
Particle size distribution using laser particle size analyzer for AG (**a**,**b**); NG (**c**,**d**) and EG (**e**,**f**).

Figure 2 shows the distribution of the particle size using laser particle size analyzer for AG, NG and EG. Artificial graphite according to laser diffraction data was characterized by a unimodal distribution shown in Figure 2a. The d10, d50 and d90 values for the filler were of 23.3, 42.9, and 64.3 µm, respectively, and the average volumetric diameter D [4.3] was of 43.2 µm. Typical values of bulk density and tapping density were 0.014 and 0.176 g/cm^3^, respectively. A similar distribution of particles was observed for natural graphite as shown in Figure 2c, the distribution parameters of which were: the d10, d50 and d90 values for the filler were of 21.6, 36.2, and 54.5 µm, respectively, and the average volumetric diameter D [4.3] was 36.9 µm. Values of bulk density and tapping density were 0.464 and 0.584 g/cm^3^, respectively. For the thermally expanded graphite obtained shown in Figure 2e, the d10, d50 and d90 values for the filler were of 22.6, 39.0, and 69.2 µm, respectively, and the average volumetric diameter D [4.3] was 52.7 µm. Typical values of bulk density and tapping density were 0.545 and 0.77 g/cm^3^, respectively. If we consider only the laser diffraction data when describing the geometry of the filler, then the sizes of the particles used practically do not differ from each other; however, SEM shows a significant difference, both in geometry and in the structure of particles, which undoubtedly influenced the results obtained.

Artificial graphite particles, as seen from the SEM image in Figure 1c, have a rounded shape, and the number of flake-like particles is insignificant. Processing of micrographs using Origin lab program gives results comparable to those obtained using laser diffraction, the bulk of the particles have a size of 20–60 µm, while there are individual particles with sizes up to 100 microns. Natural graphite, as seen from SEM image Figure 1d, has the shape of flakes, and processing of SEM data gives the characteristic dimensions: thickness 15–20 µm, and average diameter of 200–500 µm. Laser diffraction, even when using models that consider the geometry of particles when processing experimental data, gives significantly different data, but its use is justified as an additional method that makes it possible to estimate the general nature of the distribution over a very large number of particles, which is difficult when using estimates with micrographs that show predominantly large particles and the number of particles counted in this way is not large (300–500). Therefore, to obtain an adequate understanding of the geometry and size of particles and their distribution, we used several described methods.

Thermally expanded graphite is a set of thin graphene-like particles with a thickness of 10–20 atomic layers Figure 1a,b, partially retaining bonds with each other within the original particle of natural graphite, and as a result EG has a diameter 200–500 µm, width 100–300 µm and a thickness of about 35–50 µm. The distribution of particle size depending on SEM images are represented in Figure 2b,d,f.

#### 3.1.3. X-ray Diffraction

**Figure 3:**
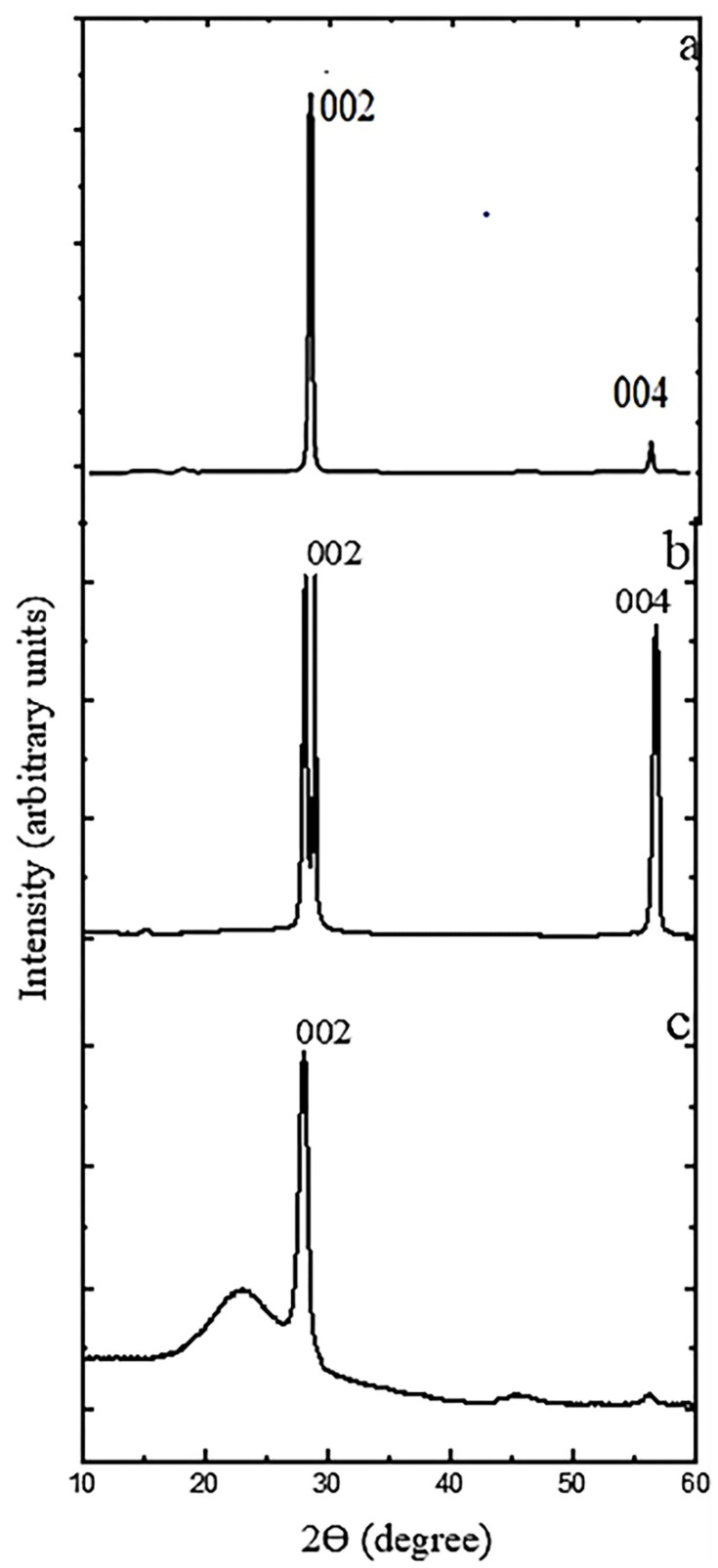
X-ray diffraction (XRD) patterns of AG (**a**), NG (**b**), EG (**c**).

Figure 3 shows the XRD pattern of used graphite materials. For NG flakes two sharp reflection peaks (Figure 3b) appear at 2θ = 28.2° and 2θ = 29°, which resulted from the diffraction of 002 planes with d-spacing of 0.302 nm and 0.310 nm, respectively, calculated with the Bragg equation. Therefore, we can assume the high crystalline structure of graphite flakes and another peak at 2θ = 55.8° corresponds to the diffraction on planes (004). After the intercalation and thermal treatment, a slightly sharp (002) peak with d-spacing of 0.321 nm appears at 2θ = 28° for EG and another peak at 2θ = 23.1° which shows that the orientation of graphite basal planes parallels to the sample plane (Figure 3c), which suggests a deformed graphite structure and thus the start of graphene sheet production. It is obvious that when K_2_Cr_2_O_7_ is oxidized, the non-carbonaceous reactant can easily be introduced into the graphite sheets, resulting in increased interplanar crystal distance. It should be noted the halo-like peak at 2θ = 23.1° should be considered as an evidence of graphene layer formation, not as the true amorphization. The surface structure has been changed in the expanded graphite which caused the weaken in the strong reflection peak at 2θ = 55.8°. For AG (Figure 3a) we see one sharp peak at 2θ = 30.5° which resulted from the diffraction of 002 planes with *d*-spacing of 0.342 nm in addition at 004 peak at 2θ = 55.5°.

The use of X-ray diffraction to determine the mean size of crystallites in crystalline materials is a convenient method. This is due to the fact that “crystallite size” is not the same as “particle size,” and X-ray diffraction is sensitive to crystallite size within the particles. Therefore, in our work we used the well-known Scherrer formula to calculate the average crystallite size *L*:

$$L=\frac{K\lambda }{\beta \cos\theta }$$

where *λ* is the X-ray wavelength in nanometer (nm). *β* is the peak width of the diffraction peak profile at half maximum height resulting from small crystallite size (FWHM) in radians, K is a constant related to crystallite shape, taken as 0.94, and the results were: L_AG_ = 33 ± 2 nm, L_NG_ = 76 ± 5 nm and L_EG_ = 6.5 ± 1 nm for AG, NG and EG, respectively.

#### 3.1.4. Raman Spectroscopy

**Figure 4:**
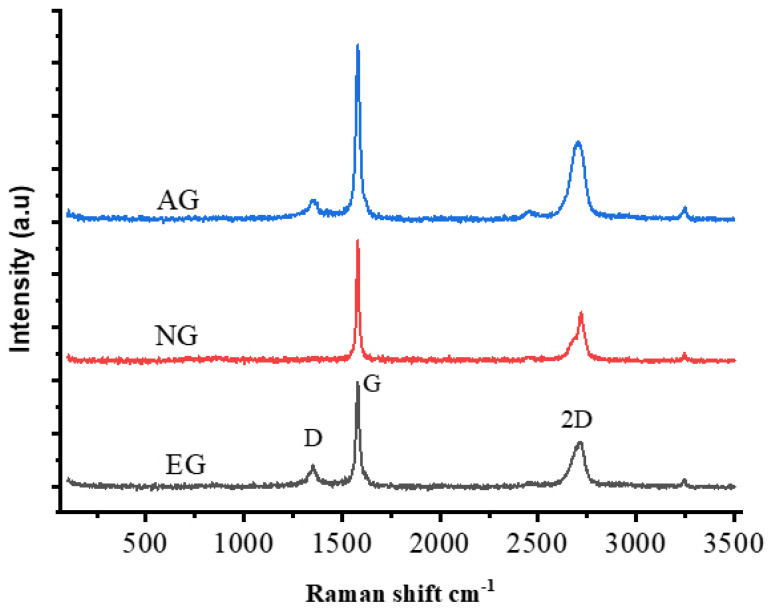
Raman spectra of AG, NG and EG.

Raman spectroscopy is useful for studying different allotropes of carbon (such as diamond, carbon nanotubes, Buckminster fullerenes, carbon nanoribbons, and so on), where the only difference between them is the relative position of their carbon atoms and the nature of their relationship to one another. The G-band appears in all sp^2^ carbon systems and is caused by the stretching of the C–C bond. Because the G-band in the sp^2^ system is highly sensitive to deformation effects, it can be used to investigate changes on a flat material surface. As shown in Figure 4, the main peaks for NG are a G peak around 1580 cm^−1^ and a 2D peak around 2714.9 cm^−1^, AG consist of a G peak around 1580.2 cm^−1^, D peak around 1350 cm^−1^ and 2D peak around 2708 cm^−1^ and EG has a G peak around 1580.09 cm^−1^, D peak around 1344.43 cm^−1^ and 2D peak around 2705 cm^−1^. The interlayer interactions at different depths within graphite cause the shoulder in the 2D peak for NG. As we can see, there is a change in the shape of the 2D peak for AG and EG caused by the reduction in van der Waals interaction between graphene layers and the increasing of the interlayer distance between these layers. The disappearance of the D peak in NG compared to AG and EG can tell us about the defective structure of those two materials as well as this peak requiring a defect in structure to activate unlike a 2D peak which does not require a defect to appear. We can use the intensity ratio D/G to know the defect level in the material, and as a result we found ID/IG = 0.103 and 0.183 for EG and AG, respectively, that tells us that AG is more defective compared to EG.

#### 3.1.5. Summary

We used three carbon materials as a heat-conducting filler: crushed artificial graphite (mark GMZ, MEZ, Moscow Russia), crushed natural graphite (GL1), and thermally expanded graphite. The use of fillers of different sizes and shapes of particles makes it possible to study the features of the formation of heat-conducting networks in a polymeric composite material based on polysulfone using a solution mixing technology. The main characteristic of graphite materials intended to use as a filler for polysulfone matrix are summarized in Table 1. It should be noted that the used materials differ significantly both in particle geometry and crystalline structure. Whereas AG consists of nearly equiaxial particles with a defective crystalline structure of graphite, NG powder particles represent flakes with perfect crystalline structure. Exfoliation of NG results in the formation of EG with individual particles containing a set of thin flakes. During exfoliation the perfect crystalline structure of NG transforms into high defective structure containing graphene layers. Observed difference in the particles geometry and crystalline structure can significantly affect both the peculiarities of the filler location in a polymer matrix and the adhesion between polymer and graphite fillers.

It should be noted that particle size determination using a laser particle size analyzer is carried out according to ISO 13320 “Particle size analysis—Laser diffraction methods”. With this method, the determined particle size is expressed as the diameter of a sphere of equivalent volume, i.e., we have flat particles reduced to spherical condition with an equivalent size determined by the diffraction of laser radiation, and this size is what the device gives us. The use of models that consider the geometry of particles when processing the results, for example, the fact that particles can be in the form of scales, still gives a significant deviation in size.

Particle sizing investigation using SEM gives inflated size values, and shifts the distribution towards large particle sizes, since during sample preparation, some of the small particles are blown away from the conductive tape, and some of the small particles are hidden under the large ones. Additionally, in the case of the SEM study we have a small amount of particles for calculating microphotographs (about 500–1000), whereas when using laser diffraction the number of analyzing particles is in the tens of thousands.

Therefore, the combination of these methods makes it possible to more fully describe the size and morphology of the particles of the fillers used, and the size and shape of particles given in Table 1 are based on the combination of the data obtained using the two methods discussed above.

### 3.2. Polysulfone-Based Composites

#### 3.2.1. Microstructure

**Figure 5:**
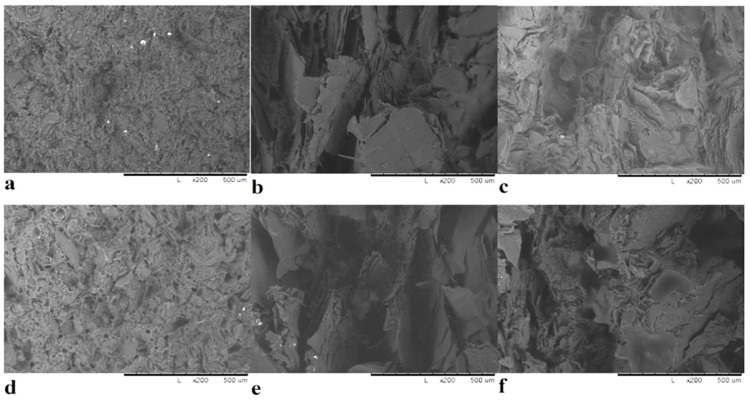
SEM micrographs of: polysulfone (PSU) + 50 wt.% AG (**a**); PSU + 50 wt.% NG (**b**); PSU + 50 wt.% EG (**c**); PSU + 70 wt.% AG (**d**); PSU + 70 wt.% NG (**e**); PSU + 70 wt.% EG (**f**).

The dispersion of artificial graphite (70–50 wt.%) in polysulfone matrix is shown in Figure 5a,d. The artificial graphite dispersion blends in well with the polymeric matrix, as can be seen. However, we detected some small pores in the composite, which could cause a flaw in its qualities; as we can see, some of these pores appeared when we split the sample to analyze its internal structure. We can see that the natural graphite sheets look like they were planted in the matrix and produced a huge agglomeration with the polymer in Figure 5b,e) for natural graphite (70–50 wt.%), in addition to pores emerging. Figure 5c,f shows the dispersion of expanded graphite (70–50 wt.% respectively) into the matrix. We can see that the expanded graphite is extensively spread in the matrix and shows highly bonded surfaces and agglomerated regions of expanded graphite with the polymer matrix, which reduces heat conduction at interfaces.

#### 3.2.2. Thermal Conductivity

The thermal conductivity was measured at different temperatures via the laser flash method using LFA 447 Nanoflash. The samples for thermal conductivity measurements were compression-molded by hot pressing (160 °C, 194 kN, 20 min) into a cylindrical shape (diameter 12.7 mm, thickness 2 mm).

The formula used to calculate the thermal conductivity is:

(1) $$\lambda =a\cdot \rho \cdot C_{p}$$

where *λ*, a, ρ and $C_{p}$ are thermal conductivity W/(m·K), thermal diffusivity mm^2^/s, material density g/cm^3^ and heat capacity j/(g·K), respectively.

**Figure 6:**
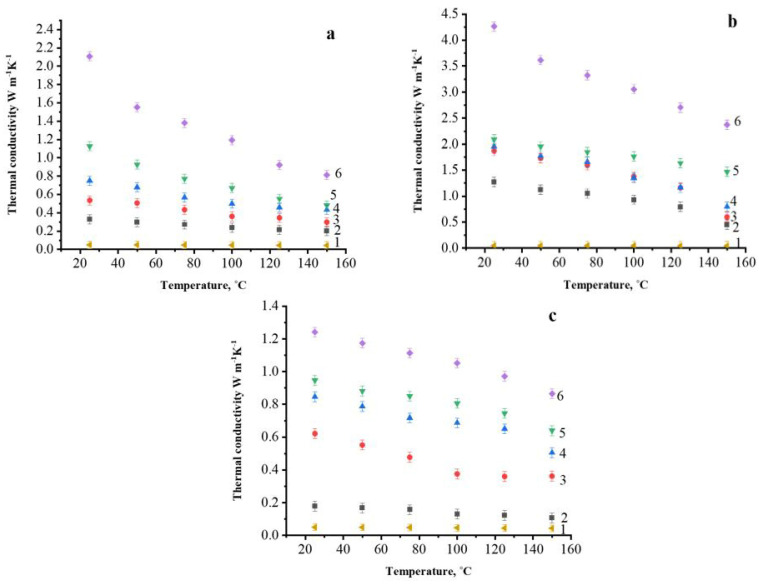
Thermal conductivity temperature dependences of PSU-based composites filled with 0 (1), 30 (2), 40 (3), 50 (4), 60 (5) and 70 (6) wt.% of AG (**a**), NG (**b**) and EG (**c**).

Figure 6 shows the thermal conductivities of a polymer filled with varying concentrations of graphite filler as a function of temperature; it should be noted that with an increase in temperature up to 150 °C, the thermal conductivity decreases by about two times for all the studied composite materials. For composites filled with NC and AG, the thermal conductivity increased when the filler concentration was increased. And as we can see that thermal conductivity of unfilled polysulfone also decreased while temperature increased.

Because of their high thermal conductivity, as well as its thermal stability and mechanical qualities, many researchers have investigated the effect of graphite materials on the thermal conductivity of polymers. Fu et al. [37] reported the results of a comparative study of thermal conductivity between different types of graphite and other materials such as Cu and AL. The results showed that the highest thermal conductivity was obtained using 44.3 wt.% graphite due to the layered structure of the graphite, which led to good heat transfer through the composite and by using 60 wt.% low-temperature EG the highest thermal conductivity was 11.24 W/m·K which is higher than thermal conductivity in our work due to the defected crystalline structure of prepared EG. Table 2 shows some of these results.

At room temperature the thermal conductivity of 70 wt.% NG is 4.26 W/m·K, which is superior to the thermal conductivity of 70 wt.% AG and 70 wt.% EG and that is due to that NG having larger crystallite sizes and a crystal structure closer to perfect single crystalline material than AG, as mentioned in Section 3.1.5. The thermal conductivity of unfilled polysulfone is about 0.05 W/m·K at 25 °C as shown in Figure 6, thus filling of polysulfone with 70 wt.% NG increases the thermal conductivity almost 100 times, whereas filling with the same amount of EG allows this to increase 20 times only. For this reason, NG typically has higher thermal and electrical conductivity. Thermal conductivity decreases when the temperature rises due to dilation in the sample, which causes changes in characteristics, however, the effect is small. Furthermore, when the temperature rises, the phonon mean free path decreases, resulting in a decrease in thermal conductivity. This is due to anharmonic scattering, which is inversely proportional to the temperature. Figure 6c shows that thermal conductivity of PSU/EG composite does not increase significantly as the filler content grows until 50 wt.% EG That is due to the big agglomerations that are formed by EG in the PSU matrix at high filler content. As EG particles have a lot of internal defects, the thermal conductivity of structure containing such agglomerates is low. Additionally, the appearance of pores causes the decrease in thermal conductivity of such composites.

**Figure 7:**
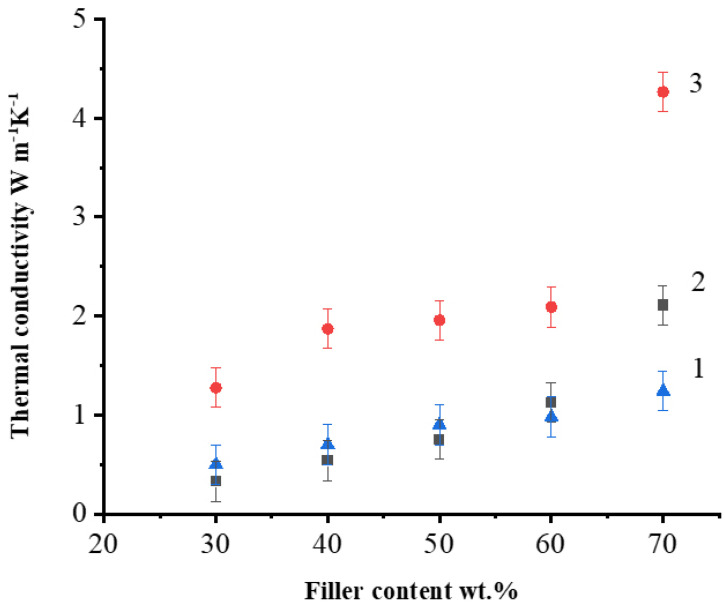
Thermal conductivity as function of filler content of unfilled PSU and PSU-based composite filled with EG (**1**); AG (**2**) and NG (**3**) at 25 °C.

Figure 7 presents the thermal conductivity of PSU-based composite filled with AG, NG, and EG at room temperature as a function of the filler content. The thermal conductivity has increased as the filler content has increased, as can be shown. With an increase in the filler concentration, the thermal conductivity values increase, however, the percolation threshold is clearly observed only for NG containing composites and it lies in the concentration range between 60 and 70 wt.% of NG. This is due to the large surface area of NG’s flakes which, as previously stated, allowed the NG particles to form well-bonding bridges with the matrix, resulting in higher thermal conductivity. The poor ability of PSU/AG and PSU/EG composites to transport heat is due to the defects in AG and EG structure, resulting in low thermal conductivity of these composites.

Figure 7 shows that with an increase in all the filler concentration, the thermal conductivity values increase, but the percolation threshold is clearly observed only for composite materials with NG at the concentration range between 60 and 70 wt.%. Thus, the most effective filler for the used method and modes of obtaining composite materials by solution technology turned out to be NG. The observed differences are due to the morphology of filler particles and its distribution within the composite material. The contact between NG particles consisting of thin flat particles with linear dimensions up to 400–500 μm occurs due to their overlap, which provides a larger contact area, and allows a more efficient percolation grid to be built than in the case of nearly equiaxial particles of AG and EG, in which the contact between is predominantly pointed.

#### 3.2.3. Mechanical Test

**Figure 8:**
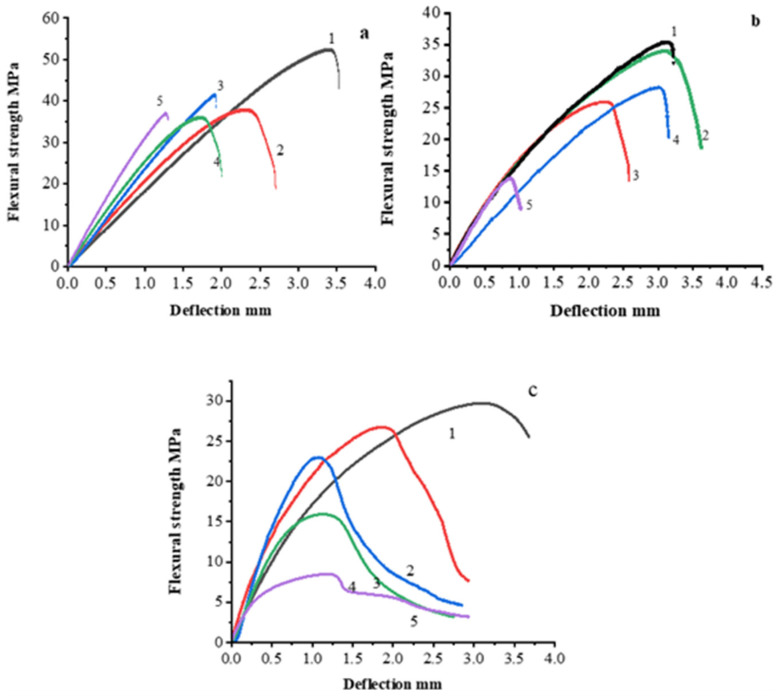
Flexural test of PSU based composites filled with 30 (1), 40 (2), 50 (3), 60 (4) and 70 (5) wt.% of AG (**a**), NG (**b**) and EG (**c**).

Three-point flexural tests were performed in accordance with ISO 178:2019 and ASTM D790 standards. The test was performed using a Zwick Roell Zwick/Roell (Group, Ulm, Germany) universal testing machine. The samples were shaped into rectangles (80 × 10 × 3 mm) using a hot press process. The flexural strength of unfilled polysulfone is about 120 MPa and the maximum achieved value of flexural strength, using 30 wt.% AG is 52.3 MPa as we can see in Figure 8a which means that the PSU/AG composites have high stiffness and this implies that the filler is well dispersed throughout the polymeric matrix, allowing mechanical stresses to be passed between the two components using contents of filler that are not very high. Then the flexural strength started to decrease as the filler content became higher. A decreasing of flexural strength for all samples was noticed while the filler content was increased, so according to that pattern in which the curves increased and subsequently decreased, we can assume that there is an enhancement in interfacial adhesion between the filler and matrix which decrease with increasing filler content because of the agglomeration forming as the filler content becomes higher. Chen Hui et. al. in their work tested the effect of graphite on the flexural strength of epoxy using different filler content from 60 wt.% to 90 wt.% and they found the flexural strength was decreased as the filler content increased and the highest achieved value of flexural strength was 52 MPa using 65 wt.% of graphite [43]. In [44] we reported that the flexural strength of PSU-based composite filled with carbon fiber up to 60 wt.% increased up to 899 ± 27 MPa, but at the further filler content increasing to 70 wt.% the flexural strength started to decrease to 811 ± 28 MPa. At a low content of carbon fiber, the flexural strength of the composite was rather low because of the significant content of the weak PSU phase. Increase of carbon fiber content resulted in an increase in fiber packing density, while a decreased interlayer between the individual fibers occurred. A decrease in the strength with a further increase in the carbon fiber content proceeded because the matrix content was too low to distribute the applied load to carbon fiber [44].

**Figure 9:**
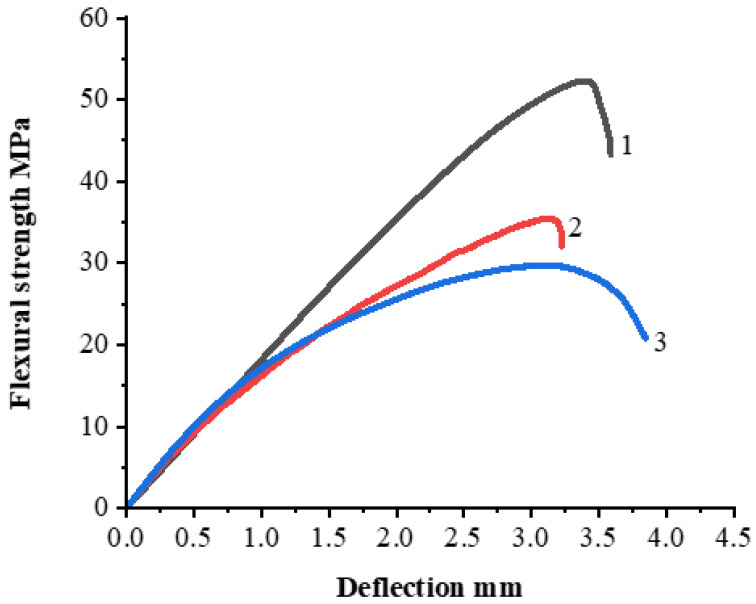
Flexural test of PSU based composites filled with 30 wt.% of EG (**1**); NG (**2**); AG (**3**).

Figure 9 shows the variation of maximum flexural strength for PSU/EG composites, and from the graph we can see that the maximum values of flexural strength were achieved from using 30 wt.%AG, NG and EG and are 52.3 MPa, 35.6 MPa and 29.7 MPa, respectively. Therefore, we can say as a result that AG has the best adhesion between the matrix and filler, which is probably a result of a defective structure of AG crystalline lattice, as indicated in Section 3.1.5. It should be noted that PSU/EG composites show the lowest level of mechanical properties between the studied composites, which in spite the crystalline structure of EG is also very defective. This can show that the used solution technology does not allow the good impregnation of the polymer into the body of EG particles, which results in a weak adhesion between polymer and filler. The variation of maximum flexural strength and young’s module is also shown in Table 3.

Polysulfone is a high-flexural-strength polymer, while graphite is a brittle material. Compounding both materials results in lower plasticity and higher stiffness and brittleness than the original properties of pure polysulfone and graphite appears to be responsible for the composite materials’ mechanical properties.

## 4. Summary and Conclusions

The design of electrical devices has tended to grow thinner and more compact in recent years. As a result, heat management has become an important factor of device and application design. Several other uses, such as electric motors and generators, heat exchangers in power generation, and automotive, confront similar problems. The addition of thermally conductive fillers to polymer-based composites has greatly increased their performance in thermal conductivity. As can be seen from the above, graphite is becoming increasingly popular for improving the thermal conductivity of composites in a variety of applications.

The solution-casting method was used in this work to prepare the PSU-based composed filled with various concentrations of fillers up to 70 wt.%. To enhance the thermal conductivity and mechanical properties, three types of graphite as a filler were used in this work. After studying the morphology and structure of the fillers we found that NG has a perfect crystalline graphite structure which made it the best filler type that improved the thermal conductivity of the composite, but both AG and EG have a defective crystalline structure which affects its properties. By studying the thermal conductivity of the composite, we found that the highest value of thermal conductivity was achieved using 70 wt.% NG and was about 4.26 W/m. K. and that, as we mentioned above, was due to the perfect crystalline structure and large aspect ratio of NG’s sheets creating a large conducting surface with the matrix, allowing heat to be transferred more easily than AG and EG.

Percolation grids of AG, as can be assumed from the SEM data, arise due to point contacts of graphite particles, while in the case of NG, contacts are formed due to particle overlap, which allows for better heat conductivity. The particles of EG form a grid due to point contacts, however, the presence of a large number of defects inside the particle significantly reduces the thermal conductivity of the composite due to the scattering of phonons on such defects. In the case of a good separation of thermally expanded graphite particles into flakes consisting of 10 to 12 layers of graphene, an increase in the thermal conductivity of such composites can be expected.

Thus, we received materials with a high level of filling, thanks to the solution technology, which allowed us to obtain a precursor for creating products. At the moment, the internal structure of materials is not optimized, and in the future work will be carried out on “architecturing” the location of filler particles, determining the optimal particle sizes, etc. The main thing that was shown in the present study was the ability to achieve high values of filling with discrete particles with the help of solution technology.

The appearance of small pores in AG structures has an impact on the material’s thermal conductivity because the porous media acts as a thermal insulator or barrier to heat removal, as well as the formation of agglomerations, which has a negative impact on thermal conductivity despite having a positive impact on mechanical properties. As we observed, EG did not produce the intended results. EG did not show the predicted outcomes since the intercalation was not complete until the end, and the large agglomerations created by EG, together with the pores in the composite, made this material a poor heat conductor. In a mechanical test which was presented in the form of a flexural test we found that the flexural strength was decreased as the filler content was increased. Using AG as a filler presented the best enhancement in the flexural test and it reached 52.3 MPa using 30 wt.% AG. Therefore, we can say that using AG gave the composite more stiffness than NG and EG while when using NG and EG as fillers, the weak adhesion between the matrix and the filler rendered the composite more brittle and plastic. We can summarize the results as follows:

1. Using solution-casting technology allows for the manufacturing of polysulfone-based composite with up to 70% filling of graphite particles and guarantees that they are distributed uniformly throughout the material. The produced press material can be used to manufacture products using the thermal pressing method, resulting in composites with a PSU matrix and a high filler content made from graphite materials;
2. With the proposed technology, the thermal conductivity coefficient of a polysulfone-based material using natural graphite as a filler was increased to 4.26 W/m K while maintaining an acceptable degree of mechanical strength;
3. Structural defect and the appearance of pores affected on both thermal and mechanical properties.

## Figures and Tables

**Table 1:** Comparison of the graphite materials structure.

Material	Shape	Scheme	Size	Crystalline Structure
AG	Nearly equiaxial	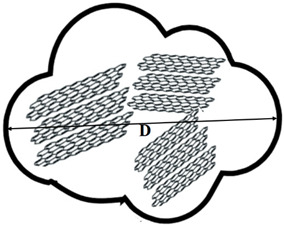	20 to 60 µm	Defective crystalline graphite structure
NG	Flakes	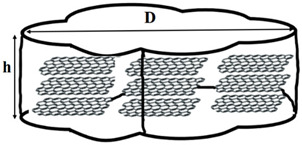	D—200 to 500 µm h—15 to 20 µm	Perfect crystalline graphite structure
EG	Set of exfoliated flakes	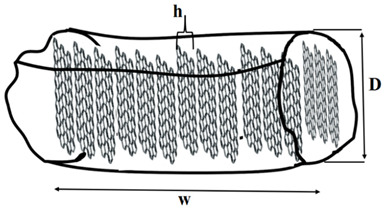	D—200 to 500 µm w—100 to 300 µm h—35 to 50 nm	Defective crystalline graphite structure containing graphene layers

**Table 2:** Thermal conductivity of graphite composite based on preview studies.

Composite	Filler Content	Thermal Conductivity W/m·K	Reference
LDPE/graphite	10 vol.%	6.5	[38]
HDPE/graphite	7 wt.%	1.59	[39]
LDPE/low-temperature Expandable graphite	50 wt.%	7.02	[40]
LDPE/low-temperature Expandable graphite	60 wt.%	11.24	[41]
Epoxy resin/graphite	44.3 wt.%	1.68	[37]
Epoxy resin/graphite	4.5 wt.%	1.0	[42]

**Table 3:** Mechanical and thermal properties of PSU-based composites filled AG, NG and EG.

Concentration wt.%	Maximum Flexural Strength MPa			E_module_ GPa			Thermal Conductivity W·m^−1^·K^−1^		
	AG	NG	EG	AG	NG	EG	AG	NG	EG
30	52.3 ± 4.5	35.6 ± 4.6	29.7 ± 5.1	7.1 ± 0.7	9.1 ± 0.8	4.8 ± 5.9	0.3 ± 0.02	1.3 ± 0.06	0.2 ± 0.03
40	41.3 ± 4.3	28.3 ± 4.8	26.8 ± 4.3	5.7 ± 0.6	6.7 ± 0.6	3.9 ± 4.9	0.5 ± 0.03	1.8 ± 0.06	0.6 ± 0.04
50	41.4 ± 4.6	34.0 ± 5.3	23.0 ± 4.8	5.2 ± 0.5	5.9 ± 0.5	4.2 ± 5.8	0.7 ± 0.05	1.9 ± 0.07	0.8 ± 0.04
60	36.5 ± 4.5	33.1 ± 4.8	16.1 ± 5.6	4.6 ± 0.6	6.0 ± 0.9	3.4 ± 4.8	1.2 ± 0.09	2.1 ± 0.08	0.9 ± 0.05
70	36.9 ± 4.8	29.1 ± 5.7	12.0 ± 5.2	4.0 ± 0.3	4.9 ± 0.8	3.9 ± 4.7	2.1 ± 0.09	4.2 ± 0.09	1.2 ± 0.09
